# Coexistence of Flexo- and Ferro-Electric Effects in an Ordered Assembly of BaTiO_3_ Nanocubes

**DOI:** 10.3390/nano12020188

**Published:** 2022-01-06

**Authors:** Kyuichi Yasui, Hiroki Itasaka, Ken-ichi Mimura, Kazumi Kato

**Affiliations:** National Institute of Advanced Industrial Science and Technology (AIST), Nagoya 463-8560, Japan; h.itasaka@aist.go.jp (H.I.); k.mimura@aist.go.jp (K.-i.M.); kzm.kato@aist.go.jp (K.K.)

**Keywords:** flexoelectric effect, ferroelectric effect, BaTiO_3_ nanocubes, ordered assembly, dielectric constant, frequency dependence, theory

## Abstract

It has been reported that the flexoelectric effect could be dominant in the nanoscale. The discrepancy between theory and experiments on the frequency dependence of the dielectric constant of an ordered assembly of BaTiO_3_ nanocubes is nearly resolved by assuming the coexistence of flexo- and ferro-electric effects. Although flexoelectric polarizations perpendicular to the applied alternating electric field contribute to the dielectric constant, those parallel to the electric field do not contribute because the magnitude of the flexoelectric polarization does not change due to the mismatch of strain at the interface of the nanocubes. On the other hand, some dielectric response is possible for the ferroelectric component of the polarization parallel to the electric field.

## 1. Introduction

To develop thinner high-performance smartphones and wearable devices, such as eyeglass-type and wristwatch-type, miniaturization and improvement of the properties of dielectric devices such as multilayer ceramic capacitors (MLCCs) are required. An ordered assembly of BaTiO_3_ nanocubes (nanocrystals) is a candidate as the main component of such devices [1,2,3,4,5,6,7,8,9,10]. The size of a BaTiO_3_ nanocube is about 15 nm, which is much smaller than the typical BaTiO_3_-based particles of about 100 nm in diameter currently used in MLCCs [5,6,11,12,13]. Furthermore, the ordered assembly of BaTiO_3_ nanocubes is formed by the self-organization process in the dip-coating method, and the defect concentration in an assembly will possibly be under control in the future [5,6,14]. Thus, the ordered assembly of BaTiO_3_ nanocubes may reduce the thickness of the dielectric layer from the current technical limit of about 500 nm to about 50 nm or less. Our research group has already successfully fabricated a mono-layer BaTiO_3_ nanocube assembly [15,16]. The dielectric constant of the ordered assemblies of 290 nm and 580 nm thickness was measured experimentally after calcinated at 400 °C for 1 h and sintered at 850 °C for 1 h in O_2_ [5,6]. There was no observable change in the structure of the ordered assembly even after calcination and sintering, except the formation of the joint at the crystal interfaces at the atomic level [5,6,17]. The measured dielectric constant was about 3800 and 2600 for 290- and 580-nm-thick assemblies, respectively, at 1 MHz at room temperature [5,6]. For both thicknesses, the amplitude of the applied alternating electric voltage was the same as 0.5 V. Thus, the amplitude of the electric field was larger for a thinner assembly; 17.24 kV cm^−1^ and 8.62 kV cm^−1^ for 290 nm and 580 nm thickness, respectively. Compared to the normal dielectric constant of a BaTiO_3_ bulk crystal (about 1600) without any domain contribution [18,19] and those of typical BaTiO_3_ thin films (lower than 1000) [20,21,22,23,24], the dielectric constants of the BaTiO_3_ nanocube assemblies are much higher. The temperature dependence of the capacitance (dielectric constant) of the ordered assembly of BaTiO_3_ nanocubes showed a very broad peak (nearly flat) with the peak maximum at around 100 °C [5].

In the previous paper [25], the authors have shown theoretically that a small tilt angle between two attached nanocubes results in compressive strain in a nanocube. As the magnitude of compressive strain decreases as the distance from the interface increases due to strain relaxation caused by dislocations, flexoelectric polarization near the interface of a nanocube is directed inward from the interface [25]. In other words, six small vectors of flexoelectric polarization near the interfaces are directed inward in a nanocube that has six interfaces [25]. Indeed, the presence of compressive stress after sintering has been experimentally confirmed in an ordered assembly of BaTiO_3_ nanocubes by the shift of the Raman peaks of BaTiO_3_ to a higher wavenumber [8]. Furthermore, it has been experimentally suggested that strain field is localized near the interfaces of nanocubes by the presence of dark regions near the interfaces in high-resolution TEM images of mono-layer BaTiO_3_ nanocubes fabricated by a drop of mesitylene solution in which BaTiO_3_ nanocubes were dispersed on a silicone substrate after sintering at 850 °C for 1 h in O_2_ [17]. The localized strain fields mean that there is a considerable strain gradient. Due to the presence of a strain gradient, electric polarization appears, which is called the flexoelectric effect [26,27,28,29,30,31,32,33]. In the previous paper [25], the magnitude of flexoelectric polarization is estimated to be one order of magnitude larger than that of ferroelectric spontaneous polarization of BaTiO_3_. It has been suggested that the dielectric response of an ordered assembly of BaTiO_3_ nanocubes is dominated by that of the flexoelectric polarization [25]. Under the assumption, the temperature dependence of the dielectric constant of an ordered assembly of BaTiO_3_ nanocubes is reproduced by numerical calculations. However, there is a considerable discrepancy between theory and experiment on the frequency dependence of dielectric constant [25]. Furthermore, in the previous paper [25], mismatch of the compressive strain at the interface of the nanocubes associated with the change in the magnitude of flexoelectric polarization in each nanocube was not considered because the strain was only implicitly considered through an anharmonic potential for flexoelectric polarization.

In the present paper, a mismatch of the compressive strain is considered. As a result, flexoelectric polarization parallel to the applied alternating electric field does not contribute to the dielectric constant. Instead, the ferroelectric component of polarization parallel to the applied alternating electric field contributes to the dielectric constant. The coexistence of flexo- and ferro-electric effects almost resolves the discrepancy between theory and experiment on the frequency dependence of dielectric constant of an ordered assembly of BaTiO_3_ nanocubes, which is an improvement compared to Ref. [25]. The coexistence of flexo- and ferro-electric effects have been discussed in many papers [34,35,36,37,38,39,40,41,42,43,44]. Catalan et al. [34] showed, by numerical calculations of the Landau-Ginzburg-Devonshire free energy, that the maximum dielectric constant of ferroelectric thin films decreases by the flexoelectric effect and that the polarization at room temperature increases by the flexoelectric effect. However, the decoupling of flexo- and ferro-electric effects in the present case of an ordered assembly of BaTiO_3_ nanocubes has never been reported. In general, the accuracy of numerical simulations based on the model of ordinary differential equations (ODE models) in the present paper may be worse compared to the first-principle calculations [37,45,46]. However, ODE models are superior to the first-principle models in that they are computationally economical and that important factors are more easily tractable [47,48]. Furthermore, in the present ODE model, the nonlinear effect is taken into account by using the nonlinear potential.

## 2. Theoretical Model

The flexoelectric polarization ($P_{3}$) is proportional to the strain gradient ($\partial \epsilon_{11}/\partial x_{3}$) as follows [33].

(1) $$P_{3}=\mu_{12}\frac{\partial \epsilon_{11}}{\partial x_{3}}$$

where $\mu_{12}$ is the flexoelectric coefficient, which is positive for BaTiO_3_ ($\mu_{12}\sim 10$ μC m^−1^ at room temperature [33]), $\epsilon_{11}$ is the transverse strain, and $x_{3}$ is the thickness direction. Near an interface of a BaTiO_3_ nanocube’s ordered assembly, the magnitude of the transverse compressive strain decreases toward the center of the nanocube. According to Equation (1), the flexoelectric polarization is directed toward the increasing direction of strain. As the compressive strain takes a negative value, the increasing direction of strain is toward the center of the nanocube from an interface. Thus, each flexoelectric polarization is directed inward from each nanocube interface. There are six vectors of flexoelectric polarization inside a nanocube because there are six interfaces.

**Figure 1:**
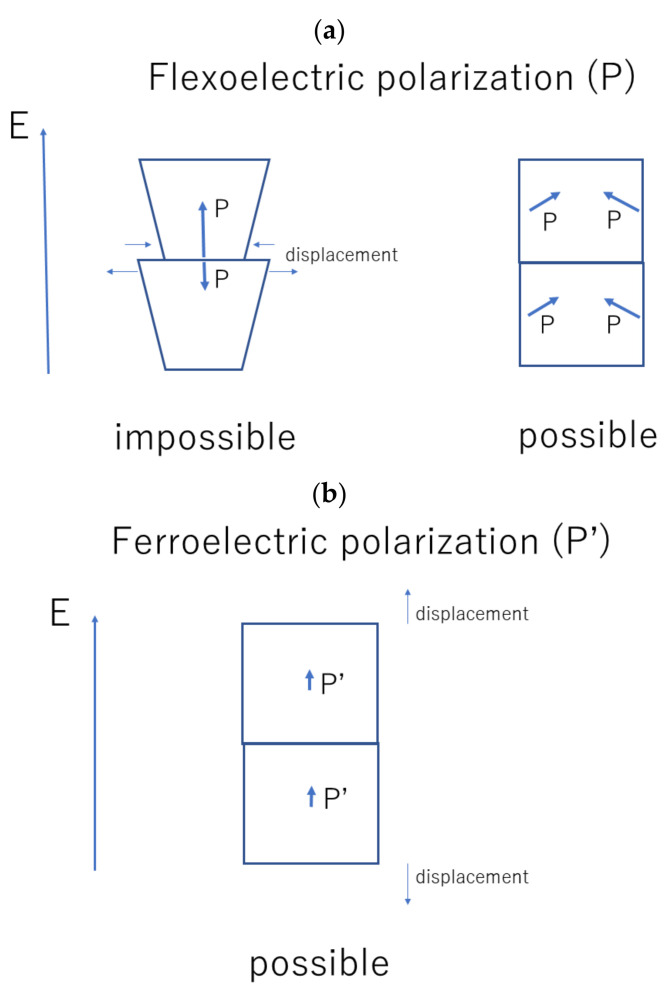
Flexo- (**a**) and ferro-electric (**b**) polarization under applied alternating electric field (E) for tightly joined BaTiO_3_ nanocubes in an ordered assembly.

Firstly, flexoelectric polarization parallel to the applied alternating electric field is considered (left of Figure 1a). The amplitude of the flexoelectric polarization tends to increase in the same direction as the applied electric field. As the amplitude of the flexoelectric polarization is proportional to the amplitude of the strain gradient (Equation (1)), the increase in amplitude of flexoelectric polarization results in an increase in the amplitude of the strain gradient. It means that magnitude of the compressive strain at the interface increases. On the other hand, the magnitude of the flexoelectric polarization in the other nanocube tends to decrease at the interface in the opposite direction to the applied electric field. It results in a decrease in the magnitude of the compressive strain at the interface. Accordingly, the change in magnitude of the compressive strain at the interface for a nanocube mismatches that of the other nanocube, as shown in the left of Figure 1a. The left of Figure 1a is exaggerated because the actual magnitude of the compressive strain at the interface is only about 0.002, and the shape of a nanocube is almost cubic even under the sliding interfaces. As the actual interfaces of nanocubes are tightly joined in an ordered assembly of BaTiO_3_ nanocubes, the mismatch of compressive strain at the interface means that the magnitude of flexoelectric polarization is parallel to the applied electric field does not change. In other words, flexoelectric polarization parallel to the applied electric field does not contribute to the dielectric constant.

Instead, some dielectric response of the polarization parallel to the applied electric field is expected, as shown in Figure 1b. There is no mismatch of strain at the interface for the change of ferroelectric polarization parallel to the applied electric field (the displacements shown in Figure 1b are possible). Thus, it is expected that some ferroelectric component of polarization parallel to the applied electric field contributes to the dielectric constant. The physical origin of the ferroelectric polarization is the polarization component in the absence of the strain gradient [49]. It is widely known that the ferroelectric polarization disappears for BaTiO_3_ nanoparticles smaller than the critical size due to the size effect [50,51,52,53,54,55,56,57]. However, in an ordered assembly of BaTiO_3_ nanocubes, the size effect is expected to be considerably weakened because surface charges (which are the origin of the size effect through the increase in free energy by depolarization) are almost compensated by each other at the interface of the nanocubes [9,10,58].

With regard to the flexoelectric polarization perpendicular to the applied electric field, shown in the right of Figure 1a, there is no mismatch of strain at the interface because the slight rotational motion of flexoelectric polarization occurs and the magnitude of the flexoelectric polarization is unchanged. The right of Figure 1a is also exaggerated because the angle of the rotation is only about 0.01 rad. Thus, the flexoelectric polarization initially perpendicular to the applied electric field fully contributes to the dielectric constant in contrast to the case of the flexoelectric polarization parallel to the applied electric field.

As four vectors of flexoelectric polarization in one nanocube are perpendicular to the applied electric field, and the other two vectors (with the ferroelectric component) are parallel to the applied electric field under the configuration of Figure 1, the averaged dielectric constant (ε) for an ordered assembly of BaTiO_3_ nanocubes is assumed to be given as follows under a constant temperature.

(2) $$\varepsilon \approx \frac{2}{3}\varepsilon_{flexo}+\frac{1}{3}\varepsilon_{ferro}$$

where $\varepsilon_{flexo}$ is the dielectric constant due to flexoelectric polarization perpendicular to the applied electric field, and $\varepsilon_{ferro}$ is the dielectric constant due to some ferroelectric polarization parallel to the applied electric field discussed in Figure 1b. It should be noted that Equation (2) is not applicable to the calculation of temperature dependence of dielectric constant because $\varepsilon_{flexo}$ and $\varepsilon_{ferro}$ are coupled in a complex way as a function of temperature. Catalan et al. [34] have shown both theoretically and experimentally that temperature dependence of dielectric constant of ferroelectric thin films becomes very broad due to the presence of strain gradient (the flexoelectric effect), such the calculation is required to compare with the experimental data of temperature dependence of dielectric constant of an ordered assembly of BaTiO_3_ nanocubes.

In the present paper, $\varepsilon_{ferro}\approx 1500$ at room temperature is assumed because the dielectric constant of an ordered assembly of BaTiO_3_ nanocubes is estimated as 1500 at room temperature, neglecting the contribution of the flexoelectric effect by the Landau-Devonshire theory for ferroelectric BaTiO_3_ nanocube-assembly with various tilt angles without domain contribution [10]. Furthermore, the frequency dependence of the dielectric constant for ferroelectric BaTiO_3_ ceramics is almost flat even without the domain contribution according to the experimental and theoretical results in References [18,19], in the frequency range of 1 kHz to 1 MHz studied in the present paper.

With regard to $\varepsilon_{flexo}$, the following equation is used as in the previous paper [25].

(3) $$\varepsilon_{flexo}\approx \frac{{\left(\Delta P\right)}_{amp}}{E_{0}}$$

where ${\left(\Delta P\right)}_{amp}$ is the amplitude of temporal variation (oscillation) of polarization in C m^−2^, and $E_{0}$ is the amplitude of the applied alternating electric field in V m^−1^. Equation (3) is a general equation for any kind of polarization when the dielectric constant is much larger than 1 [59]. ${\left(\Delta P\right)}_{amp}$ is obtained by numerical simulations of temporal variation of polarization using the following equation of angular momentum [25].

(4) $$I\frac{d^{2}\theta }{dt^{2}}=\left(p\cos\theta \right)E_{0}\sin\left(\omega_{E}t\right)-k\left(\theta -\theta_{0}\right)-\frac{\beta \left(\theta -\theta_{0}\right)}{{\left(1+\xi {\left(\theta -\theta_{0}\right)}^{2}\right)}^{2}}-\lambda \frac{d\theta }{dt}$$

where I is the (virtual) moment of inertia, θ is the angle of polarization relative to *x*-axis, t is time, p is (virtual) electric dipole moment which is related to polarization (P) as p=PV where V is volume ($V=d^{3}/6$ is assumed, where d is the size of a nanocube (=15 nm)), $\omega_{E}$ is the angular frequency of applied electric field (the electric-field vector is $\vec{E}=\left(0,E_{0}\sin\left(\omega_{E}t\right)\right))$, k is the spring constant for angular harmonic potential, $\theta_{0}$ is the equilibrium angle of polarization ($\theta_{0}=0$ is assumed), β and ξ are coefficients for angular Lorentzian potential, and λ is the angular damping constant. The constants and coefficients are determined as follows in the present paper; $\frac{k}{I}=9\times 10^{20}$(s^−2^), $\frac{\beta }{I}=3\times 10^{21}$ (s^−2^), $\xi =9\times 10^{3}$ (rad^−2^), and $\frac{\lambda }{I}=1\times 10^{14}$ (s^−1^), which are slightly different from those in the previous paper [25]. The flexoelectric polarization (P) is estimated as follows.

(5) $$P=\mu \frac{\partial \epsilon }{\partial x}\approx -\frac{\mu \cdot u_{m}}{\delta }$$

where μ is the flexoelectric coefficient (μ≈10 μC m^−1^ is assumed for BaTiO_3_ at room temperature [33]), ∂ϵ/∂x is the strain gradient, $u_{m}$ is the misfit strain at the interface of nanocubes ($u_{m}=-0.002$ is assumed for an ordered assembly of BaTiO_3_ nanocubes [25]), and δ is the width of the strain region (δ≈d/2 is assumed [25]). Thus, the flexoelectric polarization is estimated as P≈2.67 C m^−2^, which is one order of magnitude larger than the spontaneous polarization of BaTiO_3_ [25].

**Figure 2:**
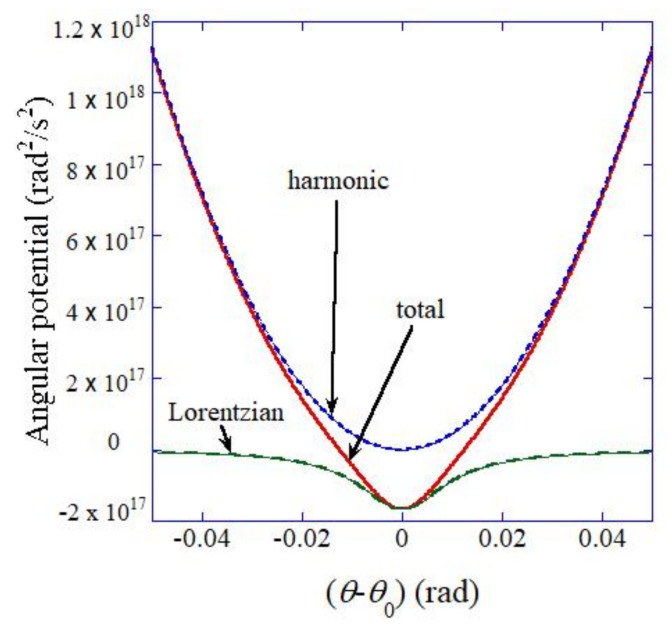
Assumed angular potential (Equation (6)).

In Equation (4), the following nonlinear angular potential ($U_{\theta }$) is used [25].

(6) $$U_{\theta }=\frac{k}{2I}{\left(\theta -\theta_{0}\right)}^{2}-\frac{\beta }{2\xi I}\times \frac{1}{\left(1+\xi {\left(\theta -\theta_{0}\right)}^{2}\right)}$$

where the first term is the harmonic potential, and the second term is the nonlinear Lorentzian attractive-potential. The assumed angular potential is shown in Figure 2, which is slightly different from that in the previous paper [25].

## 3. Results and Discussion

**Figure 3:**
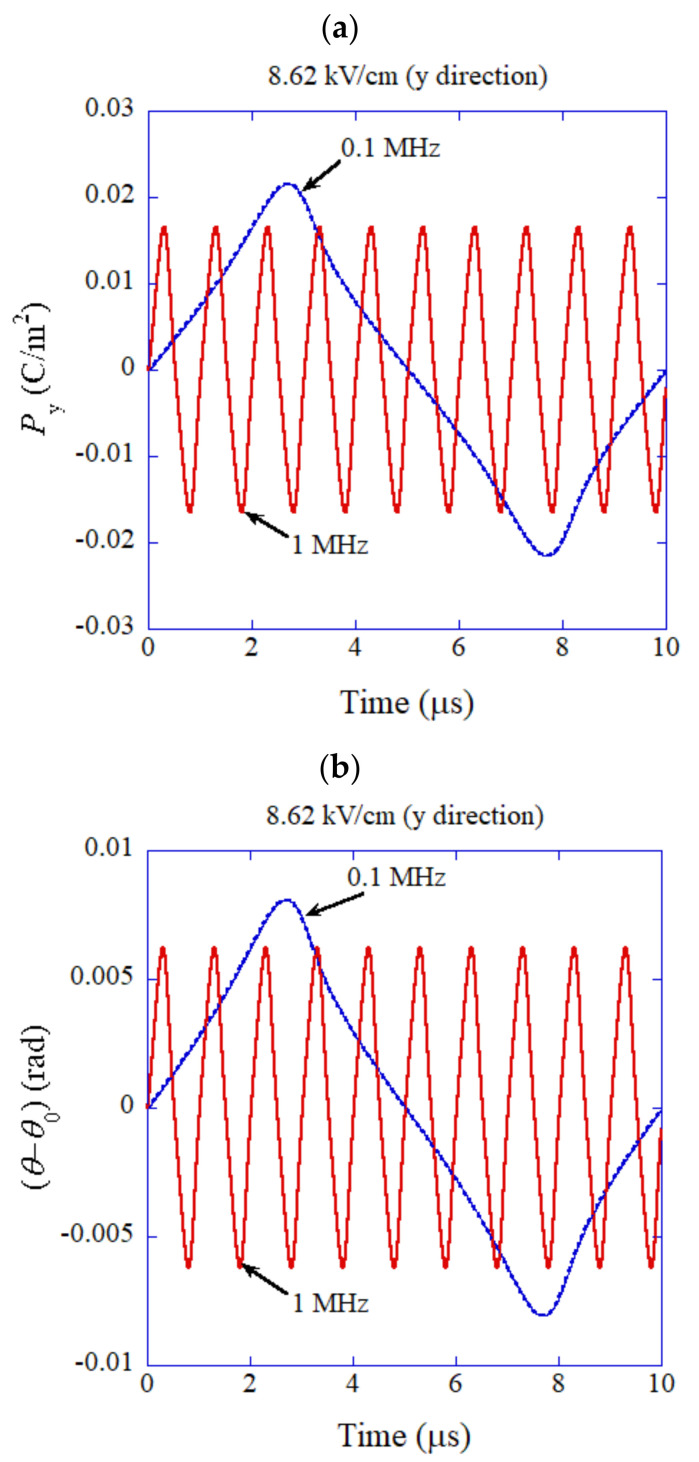
The results of numerical simulations for flexoelectric polarization initially perpendicular to the applied alternating electric field (*y*-direction) as a function of time. (**a**) The component of polarization parallel to the applied electric field (*y*-direction). (**b**) The angle (θ) of polarization ($\theta_{0}=0).$

The results of numerical simulations for flexoelectric polarization initially perpendicular to the applied alternating electric field are shown in Figure 3 as a function of time for the frequencies of 0.1 MHz and 1 MHz. Due to the nonlinear potential assumed in the numerical simulations shown in Figure 2, the waveforms shown in Figure 3 deviate significantly from the sinusoidal function, especially for 0.1 MHz. The amplitude of oscillation for the component of polarization ($P_{y}$) parallel to the applied alternating electric field is larger for lower frequency because of the nonlinear potential and less damping due to smaller angular velocity. As a result, the dielectric constant is larger for a lower frequency.

**Figure 4:**
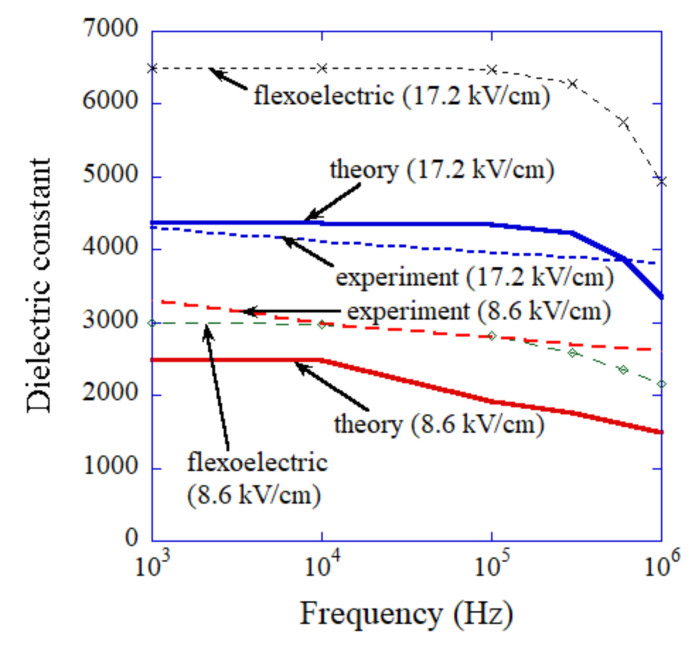
Dielectric constant as a function of frequency. The numerical results on dielectric constant solely by flexoelectric polarization are also shown. The experimental data are also shown for comparison.

The results of the numerical simulations are summarized in Figure 4 with regard to the frequency dependence of dielectric constant for the amplitudes of applied alternating electric-field of 17.24 kV cm^−1^ and 8.62 kV cm^−1^. For comparison, the experimental data of dielectric constant as a function of frequency are also shown for the thickness of assembly of 290 nm (17.24 kV cm^−1^) and 580 nm (8.62 kV cm^−1^). The results of numerical simulations for dielectric constant solely by flexoelectric polarization ($\varepsilon_{flexo})$ are also shown in Figure 4. The numerical results (ε in Equation (2)) agree relatively well with the corresponding experimental data. On the other hand, $\varepsilon_{flexo}$ deviates significantly from the experimental data for 17.24 kV cm^−1^. Thus, it is concluded that the theoretical results of the dielectric constant as a function of frequency agree considerably better with the experimental data by considering the coexistence of the flexo- and ferro-electric effect in an ordered assembly of BaTiO_3_ nanocubes.

## 4. Conclusions

It is shown that the flexoelectric polarization parallel to the applied alternating electric field does not contribute to the dielectric constant due to the mismatch of the strain at the interface of the nanocubes. On the other hand, some ferroelectric components of polarization parallel to the applied alternating electric field are expected to contribute to the dielectric constant. With regard to flexoelectric polarization initially perpendicular to the applied alternating electric field, it contributes to the dielectric constant by the rotational motion of the polarization because there is no mismatch of strain at the interface in this case. The results of numerical simulations on the frequency dependence of the dielectric constant under the assumption of the coexistence of flexo- and ferro-electric effects agree considerably better with the experimental data. It confirms the importance of the flexoelectric effect in an ordered assembly of BaTiO_3_ nanocubes.

## Data Availability

The data presented in this study are available within the article.
